# Experimental Evolution Reveals Interplay between Sch9 and Polyploid Stability in Yeast

**DOI:** 10.1371/journal.pgen.1006409

**Published:** 2016-11-03

**Authors:** Yi-Jin Lu, Krishna B. S. Swamy, Jun-Yi Leu

**Affiliations:** 1 Department of Life Sciences and Institute of Genome Sciences, National Yang-Ming University, Taipei, Taiwan; 2 Institute of Molecular Biology, Academia Sinica, Taipei, Taiwan; Harvard University, UNITED STATES

## Abstract

Polyploidization has crucial impacts on the evolution of different eukaryotic lineages including fungi, plants and animals. Recent genome data suggest that, for many polyploidization events, all duplicated chromosomes are maintained and genome reorganizations occur much later during evolution. However, newly-formed polyploid genomes are intrinsically unstable and often quickly degenerate into aneuploidy or diploidy. The transition between these two states remains enigmatic. In this study, laboratory evolution experiments were conducted to investigate this phenomenon. We show that robust tetraploidy is achieved in evolved yeast cells by increasing the abundance of Sch9—a protein kinase activated by the TORC1 (Target of Rapamycin Complex 1) and other signaling pathways. Overexpressing *SCH9*, but not *TOR1*, allows newly-formed tetraploids to exhibit evolved phenotypes and knocking out *SCH9* diminishes the evolved phenotypes. Furthermore, when cells were challenged with conditions causing ancestral cells to evolve aneuploidy, tetraploidy was maintained in the evolved lines. Our results reveal a determinant role for Sch9 during the early stage of polyploid evolution.

## Author Summary

Polyploidy is frequently observed in eukaryotes, including in human liver cells and cancer. Evolutionary studies also suggest that polyploidy has contributed to species diversification and novel adaptation in fungi, plants and animals. However, artificially-constructed polyploids often display chromosome instability and quickly convert to aneuploids. This phenomenon conflicts with observations that many species derived from ancient genome duplications have maintained the extra number of chromosomes following polyploidization. What happened during the early stages of these polyploidy events that stabilized the duplicated genomes? We used laboratory evolution experiments to investigate this process. After being propagated in a rich medium at 23°C for 1000 generations, newly-constructed tetraploid yeast cells had evolved stable genomes. In addition, evolved cells acquired resistance to stresses specific to tetraploids and exhibited a more diploid-like transcriptome profile. Further analyses indicated that Sch9—the functional ortholog of mammalian S6 kinase involved in protein homeostasis, G1 progression, stress response and nutrient signaling—contributed to the evolved phenotypes. Evolved cells increased the protein abundance and stability of Sch9. Reconstitution experiments showed that overexpression of *SCH9* enabled ancestral cells to display the evolved phenotypes and eliminating *SCH9* diminished the evolved phenotypes. Finally, we show that evolved cells were able to maintain their genomes even under a condition that causes newly-formed tetraploids to evolve aneuploidy. Our results reveal that at the early stages after genome duplication, stable polyploidy can be achieved by fine-tuning a conserved key regulator coordinating multiple cellular processes.

## Introduction

Polyploid organisms that contain more than two sets of genomes are commonly found in natural eukaryotic populations (reviewed in [1]). Variation in genome size can impact on gene expression [2], growth rate [3], and cell morphology [4]. In addition, polyploidy allows organisms to develop functional novelties using the extra copies of genetic material, which has been suggested to play an important role in organismal adaptation [5–8]. At the population level, polyploidization (or events of whole genome duplication) immediately poses a reproductive isolation barrier between the newly-formed and parental populations. Such events have been speculated to accelerate the speciation process, especially in plants [9].

However, newly-formed polyploid genomes can perturb cellular homeostasis and are also intrinsically unstable [10, 11]. Chromosome instability and rapid genome repatterning are often observed in newly-formed polyploid cells [12–15]. Under several conditions, the polyploid state may be transient and quickly degenerates into aneuploidy or diploidy [16–18]. Nevertheless, in some cases the duplicated genomes are maintained and genomic reorganizations occur at a protracted rate [19–22], raising the possibility that under certain circumstances polyploid cells can experience different types of selection before evolving stabilized duplicated genomes. However, little is known about the early stage of polyploidy evolution and the mechanisms underlying stabilized polyploid cells are poorly understood [23].

The budding yeast *Saccharomyces cerevisiae* is an excellent model organism for studying genome evolution. Yeast cells alternate between haploid and diploid states in their normal life-cycle [24], and polyploid yeast cells are commonly found in human-associated environments, including bread-making [25], lager-brewing [26–31], and amongst clinical specimens [32]. It has also been reported that many yeast strains isolated from natural populations at Evolution Canyon, Israel, are polyploid [33]. Moreover, genome structure data indicate that a whole genome duplication event occurred about 100–150 million years ago during the evolution of *Saccharomyces* yeasts [34], and *S*. *cerevisiae* is derived from this polyploid lineage [35–37]. These observations suggest that yeast cells are able to evolve a stable polyploid genome under certain conditions and stabilized polyploids may contribute to their adaptation to diverse environments.

Previously, laboratory-synthesized tetraploid yeast cells have been used to characterize their cell physiology. Several genes involved in the cell cycle and cytoskeletal organization are differentially expressed in tetraploid cells, suggesting that cells regulate these genes to accommodate the altered ploidy [2]. In addition, tetraploid cells lose viability more rapidly in stationary phase [38] and are more sensitive to mutations affecting homologous recombination, sister chromatid cohesion and mitotic spindle function [16, 39]. These results clearly demonstrate that the newly-formed polyploid genome imposes an intrinsic stress (and fitness cost) on the cells, even under conditions deemed non-stressful for diploid cells.

In addition to physiological characterization, artificially-constructed yeast tetraploids have also been studied by subjecting them to laboratory evolution experiments [7, 40, 41]. Many tetraploids quickly became aneuploid or diploid within a few hundred generations. However, in these experiments, tetraploid cells were grown either in stressed conditions or at 30°C, which is suitable for diploid cells but still stressful for tetraploid cells [16]. Under such conditions, cells with reorganized genomes are able to gain a fitness advantage and take over the population quickly [40]. Although these studies provide valuable information about how polyploidy accelerates environmental adaptation and how polyploid genomes reorganize under stress, questions remain as to whether and how a cell can adapt to a polyploid genome without drastic genome reorganization.

To gain insight into the mechanisms underlying polyploidy assimilation, we evolved newly-formed tetraploid cells in rich media at 23°C for 1000 generations and analyzed the evolved lineage. Being less stressful to the tetraploid cells, only a small difference in fitness has been observed between tetraploids and diploids under these growth conditions [16]. We hypothesized that under these conditions, small-scale mutations fixing the intrinsic defects of polyploid genomes may have a chance to survive in populations, instead of being immediately outcompeted by cells with reorganized genomes. Indeed, not only did most of our evolved strains remain tetraploid, they also exhibited higher fitness even under stressful conditions, suggesting that the evolved cells had resolved the problems associated with having newly-formed polyploid genomes and, thereby, became more physiologically robust. Further analyses revealed that Sch9—the functional ortholog of mammalian S6 kinase involved in protein homeostasis, G1 progression, stress response and nutrient signaling [42–45]—contributed to the evolved phenotypes. Finally, we show that our evolved cells could stably maintain the tetraploid genome when they encountered a more stringent condition under which the ancestral cells quickly became aneuploid, suggesting that evolved cells were able to take different evolutionary paths under stress conditions.

## Results

### Newly-formed tetraploid genomes are stably maintained in evolved yeast cell lines

Organisms with stable polyploid genomes are commonly observed in natural populations or during evolution [1]. However, our knowledge of the early stage of polyploidy evolution remains limited. To understand how a newly-formed tetraploid genome is stabilized, we constructed **a**/**a**/α/α tetraploid *S*. *cerevisiae* strains (the ancestral strains) and used them to set up laboratory evolution experiments. In these ancestral strains, the *SPO11* gene essential for meiotic recombination was deleted to prevent the cell from generating viable meiotic products (i.e. diploid progeny in our experiment) [46]. Eight lines of tetraploid cells (4N) and eight lines of control diploid cells (2N) were propagated in regular YPD medium at 23°C with daily dilution (see Materials and Methods for details). After evolving for around 1000 generations, the evolved populations were analyzed.

**Fig 1:**
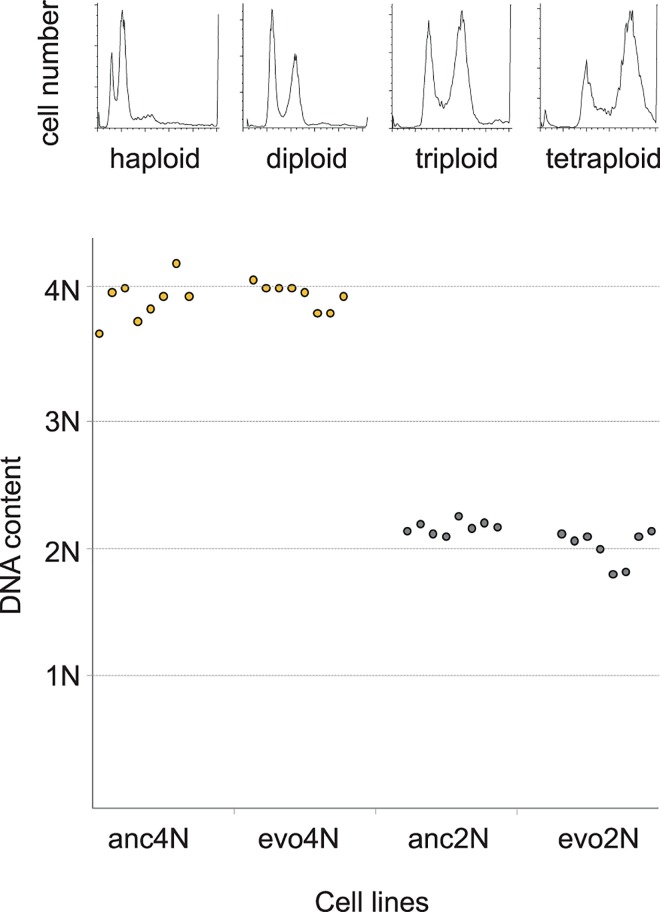
Flow cytometry analysis indicates that the DNA content of evolved cultures is similar to that of ancestral cells. The upper panel shows flow cytomery data of the DNA content in standard haploid, diploid, triploid and tetraploid populations. The mean fluorescence intensity (in arbitrary units) of G1 cells in each strain was used as the reference points (1N, 2N, 3N, and 4N shown in the lower panel). The lower panel shows the DNA content of ancestral and evolved cultures. Newly-constructed isogenic diploid (grey) and tetraploid (orange) cells were propagated in YPD medium at 23°C with daily dilutions. Cell cultures at generation zero (anc2N and anc4N) and 1000 (evo2N and evo4N) were analyzed using flow cytometry.

In previous evolution experiments, all tetraploid lines became aneuploid or diploid within a few hundred generations of evolution [7, 40, 41]. Thus, we first examined the genome size of our evolved populations by flow cytometry. The results showed that the genome sizes of evolved tetraploid populations remained similar to that of ancestral populations (Fig 1), indicating that there was no global genome reduction in our evolved lines.

### Most evolved tetraploid clones remain euploid

**Fig 2:**
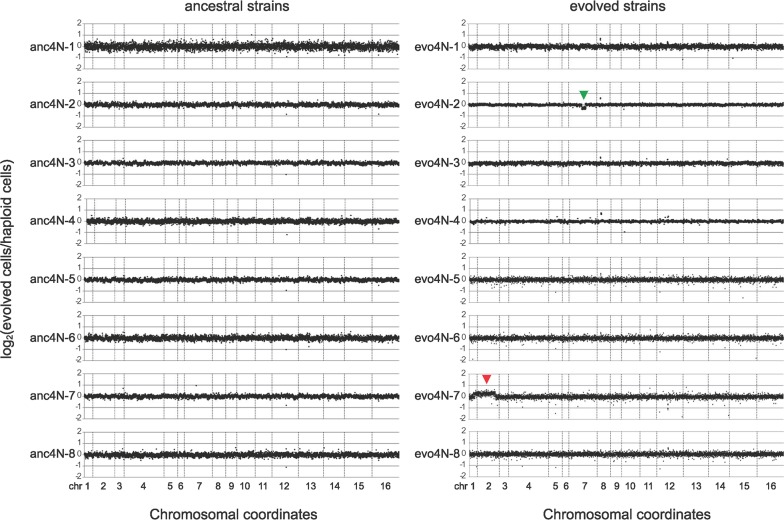
Array-based comparative genome hybridization (aCGH) analysis reveals that most evolved tetraploid clones remain euploid. Total genomic DNA was isolated from ancestral clones and the representative clones from individual evolved cultures and subjected to aCGH. Genomic DNA of a standard haploid strain was used as the reference. The ratios of signal intensities between tetraploid and reference genomic DNA were log_2_-transformed. Each dot represents a gene, and all genes of the 16 yeast chromosomes were plotted. Of the evolved clones, only evo4N-2 carried a 154-kb deletion on chromosome VII (indicated by a green arrowhead) and evo4N-7 contained an extra chromosome II (indicated by a red arrowhead). The remaining clones did not have large-scale deletions or duplications.

To characterize the evolved phenotypes, five single colonies were isolated from each 1000-generation culture and measured for their fitness using the competitive relative fitness assay. The colonies with highest fitness from each population were then selected for further examinations. Clones evo4N-1 to evo4N-8 were taken as representative of evolved tetraploid cultures and evo2N-1 to evo2N-8 represented evolved diploid cultures. In the flow cytometry experiments, we observed small fluctuations in the DNA content between different cell cultures, indicating that evolved cells might have become aneuploid or changed their mitochondrial DNA copy number. In order to determine the precise chromosomal composition of evolved tetraploid cells, we analyzed their genomes by array-based comparative genomic hybridization (aCGH) using *S*. *cerevisiae* oligonucleotide microarrays. This method allowed us to detect copy number variation at the gene level. The aCGH results revealed that the evo4N-2 clone carried a 154-kb segmental deletion on the right arm of chromosome VII (between *YGR039W* and *YGR119C*) and the evo4N-7 clone gained an extra chromosome II (Fig 2). For the remaining evolved clones, copy number variations could be observed in some individual genes or telomeric regions, but no large-scale segmental changes were detected. These results, combined with the flow cytometry data, confirmed that most evolved clones remained euploid with four genome copies.

### Evolved tetraploid clones become robust to high temperatures

**Fig 3:**
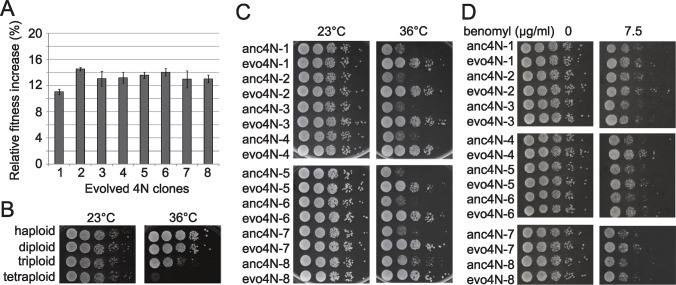
Evolved tetraploid clones have improved fitness even under stress conditions. (A) Evolved tetraploids have increased growth rates at 28°C. The growth rates of individual clones were measured using a flow cytometry-based competitive assay (see Materials and Methods). The relative fitness increase indicates the difference between the evolved and ancestral clones. Three replicates of the fitness measurement were performed. Error bars represent the standard deviation. (B) Polyploid cells are sensitive to high temperature. Cell cultures of different ploidy were serially-diluted and spotted onto YPD plates. The plates were then incubated at 23°C or 36°C until colonies were easily observed. (C) Robust growth at 36°C is detected in the evolved tetraploid clones, but not in the ancestral clones. (D) Evolved tetraploids display various levels of improvement in fitness when grown on plates containing the spindle formation inhibitor benomyl (7.5 μg/ml) at 23°C.

Although our laboratory evolution experiment was conducted at 23°C, we observed an 11%–15% increase in the fitness of evolved tetraploid clones when grown at 28°C (Fig 3A). It has been shown that polyploid yeast cells are highly sensitive to elevated temperatures, probably due to compromised cellular functions and/or increased genome instability (Fig 3B) [16]. We hypothesized that if compromised cellular functions were already compensated in the evolved tetraploid cells, then the evolved cells should become more tolerant to heat stress. We challenged the ancestral and evolved strains by growing them at 36°C. While the ancestral tetraploid strains could barely grow at this temperature, all evolved tetraploid strains exhibited much improved fitness (Fig 3C). In contrast, no significant improvement was observed in evolved diploid strains (S1A Fig), suggesting that the adaptive phenotype is tetraploid-specific.

### Evolved tetraploid clones increase their resistance to benomyl

Tetraploid cells are more sensitive to mutations affecting mitotic spindle functions and the microtubule depolymerizing drug benomyl. This defect is probably caused by geometric constraints in scaling the mitotic spindle and may have profound effects on genome stability [16]. Since tetraploid genomes were stably maintained in the evolved clones, it is possible that evolved cells have partially overcome the spindle defect. We tested this idea by challenging ancestral and evolved tetraploid clones with 7.5 μg/ml of benomyl. All evolved tetraploid clones displayed various levels of improvement in fitness when grown on benomyl-containing plates at 23°C (Fig 3D). However, the benomyl resistance of evolved tetraploids was still much lower than that of diploids, suggesting that the spindle defect in tetraploids was not completely resolved.

### Transcriptome data reveals that protein homeostasis machineries are adjusted in the evolved cells

In order to gain mechanistic insight into the adaptive phenotype, differences in whole-genome expression between ancestral and evolved cells were measured using microarrays. Since all the evolved tetraploid clones showed similar fitness at 36°C, one of the evolved clones, evo4N-4, was compared with its own ancestor, anc4N-4, in detail. Interestingly, when growing at 23°C, only 16 genes were differentially expressed (n-fold change ≥ 2, p < 0.005) between evolved and ancestral tetraploids. In contrast, 85 genes were up-regulated and 159 genes were down-regulated in evo4N-4 (n-fold change ≥ 2, p < 0.005) compared with anc4N-4 at 36°C (S1 Table). These changes were tetraploid-specific since only one misregulated gene was observed when the transcriptomes of ancestral and evolved diploids were compared under the same conditions (S1 Table). In addition, these changes were not caused by gene copy number variation in the evolved genome. These data corroborate our fitness measurements in which only minor differences were observed between evolved and ancestral tetraploids at 23°C, but drastic physiological changes were detected at 36°C.

**Fig 4:**
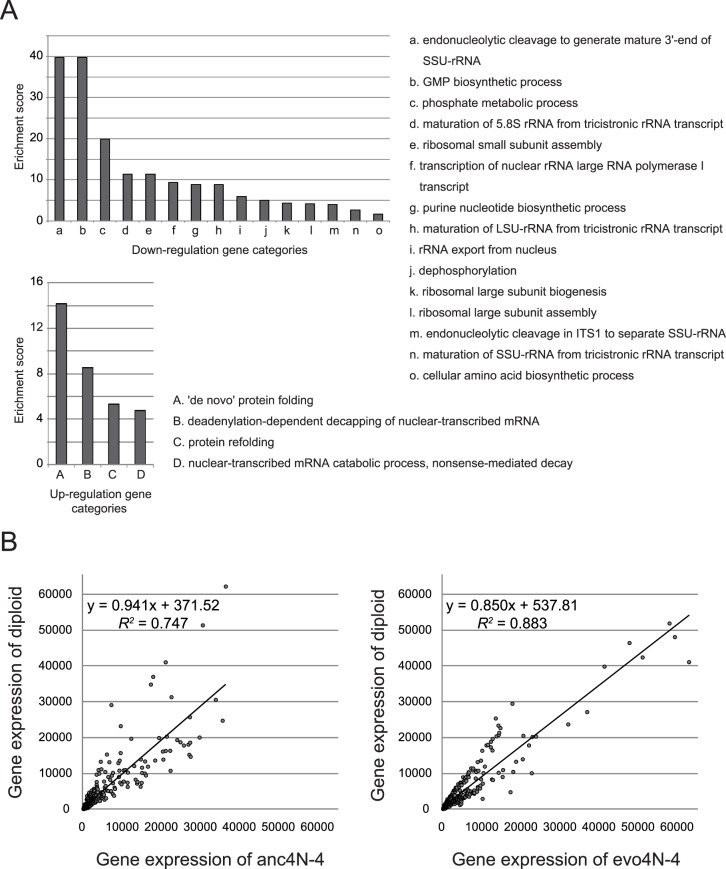
Transcriptome data reveals that protein homeostasis machineries are adjusted in the evolved clone. (A) Biological processes enriched in the down-regulated and up-regulated genes of the evo4N-4 clone at 36°C. Whole-genome gene expression levels of anc4N-4 and evo4N-4 were measured using microarrays and differentially-regulated genes were analyzed using GO analysis. The enrichment score was calculated by dividing the proportion of differentially-regulated genes classified into the indicated category by the proportion of those in the genome. Down-regulated genes are predominantly involved in rRNA processing, ribosome biogenesis and translation. Up-regulated genes are enriched in the processes of protein folding and mRNA processing. (B) The tetraploid cells have become physiologically diploid-like in their gene expression after evolution. Genes with at least 2-fold changes between anc4N-4 and evo4N-4 were selected for the comparison, and a higher correlation in expression levels was observed between ancestral diploids and evolved tetraploids (Fisher’s z-transformation, Z-value = 7.435, p-value = 1.178 × 10^−13^). A comparison using all gene expression data is shown in S2 Fig.

Further gene ontology analysis of the differentially-expressed genes revealed that a few cellular processes were strongly enriched (Fig 4A and S2 Table). Amongst the up-regulated genes, those involved in protein folding and mRNA processing were two major enriched categories. In addition, a substantial fraction of the up-regulated genes (14/85 = 16%) were involved in mitochondrial function even though this category was not enriched. The down-regulated genes were predominantly enriched for cellular processes involved in rRNA processing, ribosome biogenesis and translation, together comprising almost two-thirds of the down-regulated genes (105/159 = 66%). The data on up- and down-regulated genes suggests that excessive protein and mRNA production might represent a major intrinsic stress for newly-formed tetraploid cells and that protein homeostasis machineries were reorganized in the evolved cells to cope with this stress.

Next, we compared evo4N-4 and anc4N-4 individually with an ancestral diploid clone to investigate how ploidy influences gene expression patterns under different conditions. Intriguingly, some genes (e.g., *HSP42*, *SSA4*, *RPL39* and *RPL43A*) showed considerable differences between anc4N-4 and diploids at 36°C, but these differences vanished between evo4N-4 and diploids. Since diploidy represents a steady state in yeast cells [24], this observation raised the possibility that after evolution, the tetraploid cells could have adopted a diploid-like gene expression pattern while still maintaining a tetraploid DNA content. We selected all the genes that exhibited at least 2-fold changes between anc4N-4 and evo4N-4, and compared their expression levels between tetraploids and diploids. A higher correlation was indeed observed in the evolved tetraploid-isogenic diploid comparison (linear regression, evo4N-4 vs 2N, *R*^2^ = 0.883; anc4N-4 vs 2N, *R*^2^ = 0.747, Fig 4B), suggesting genome assimilation might have occurred during the evolution. The robustness of increased gene expression similarity between evolved tetraploids and isogenic diploids was further confirmed by performing a Fisher’s z-transformation. The transformation showed the difference between the correlation coefficient values to be statistically significant (Z-value = 7.435, p-value = 1.178 × 10^−13^). A similar trend was also observed when the whole-genome expression patterns were compared (S2 Fig). However, the difference is much smaller since most genes were not differentially expressed (linear regression, evo4N-4 vs 2N, *R*^2^ = 0.935; anc4N-4 vs 2N, *R*^2^ = 0.911).

### The protein abundance of Sch9 is increased in most evolved clones

**Fig 5:**
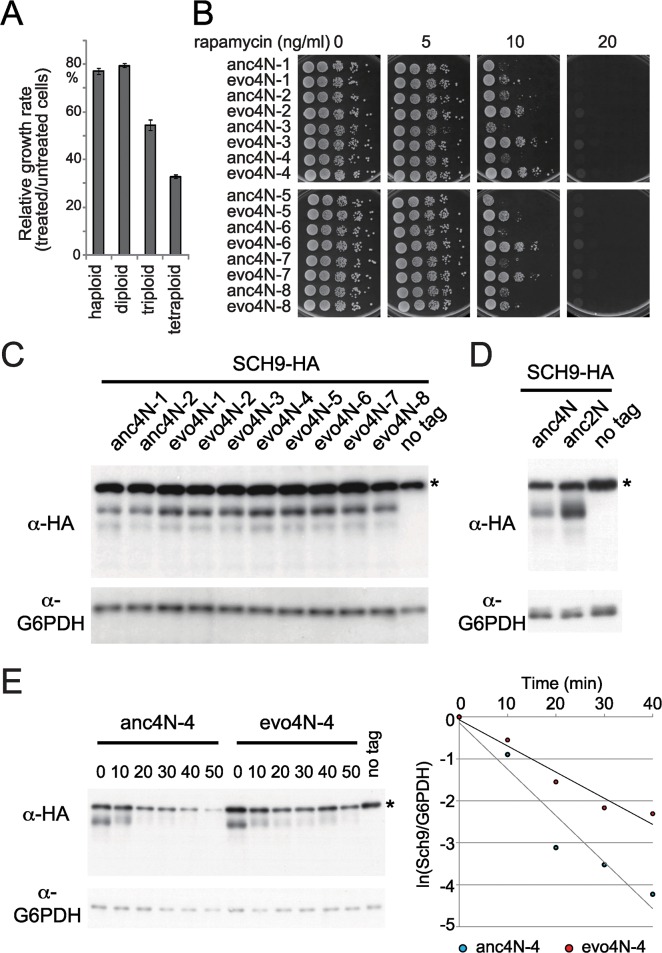
Tetraploid cells increase the protein abundance of Sch9 and resistance to an inhibitor of TORC1 networks after evolution. (A) Polyploid cells are more sensitive to rapamycin. Growth rates of cells with different ploidy were measured in liquid YPD medium with or without 10 ng/ml of rapamycin. Relative growth rates of each strain in rapamycin-containing medium were normalized to that of medium lacking the drug. Three replicates of the fitness measurement were performed. Error bars represent the standard deviation. (B) Most evolved tetraploid clones increase their resistance to rapamycin. Ancestral and evolved cells were serially diluted and spotted onto YPD plates containing different concentrations of rapamycin (0, 5, 10 and 20 ng/ml). The plates were then incubated at 23°C until colonies were easily observed. (C) Total protein was extracted from cells with or without HA-tagged *SCH9*. Immunoblotting analysis showed that the abundance of Sch9 protein was increased more than 1.5-fold in most evolved clones. G6PDH was used as the internal control. For the HA-tagged construct, three independent transformants from ancestral or evolved clones were tested and consistent patterns were observed between clones. (D) The relative abundance of Sch9 in ancestral tetraploids is about 1.8-fold lower than that in ancestral diploids. The ratio has been corrected for the difference in copy number (i.e., only one of four copies of *SCH9* is tagged with HA in tetraploids, but half of the copies are tagged in diploids). (E) The protein stability of Sch9 is enhanced in the evolved tetraploid cells. Log-phase cells were treated with cycloheximide to stop protein synthesis and collected at different time points. Total cell protein was extracted and examined by Western blot. The right panel shows quantitative data of the Western blot. The 50-min time point was not included since the signal in ancestral cells was already undetectable. All the Sch9/G6PDH ratios were normalized to the first time point data (0 min) of the same strain. Protein half-life was calculated using the change of relative protein intensity after the cycloheximide treatment (see Materials and Methods). The asterisk indicates non-specific hybridization that appears in both HA-tagged and non-tagged strains.

How do evolved tetraploid cells adjust their protein homeostasis to relieve the stress caused by increased ploidy? The conserved Target of Rapamycin Complex 1 (TORC1) network has been known to coordinate nutrient signaling, stress responses and ribosome biogenesis to regulate cell physiology [47–50], raising the possibility that it may influence polyploid cells similarly. A recent study has also indicated that TORC1 is involved in cell size control, and polyploidy is often associated with increased cell size [2, 51]. Interestingly, when our differentially-expressed gene list from evo4N-4 was compared with the data from the cells with altered TORC1 networks [52], more than one third of the genes overlapped (S3 Table). This prompted us to investigate whether TORC1 is involved in the evolved tetraploid phenotypes. Yeast cells with different ploidy levels were first tested for their sensitivity to rapamycin, a drug that interferes with TORC1 functionality. We observed that cells with higher ploidy indeed displayed higher sensitivity to rapamycin even when grown at 23°C (Fig 5A). However, when both evolved and ancestral cells were inoculated on rapamycin-containing plates, many evolved tetraploid clones (including evo4N-4) exhibited much improved growth (Fig 5B). In contrast, no significant improvement was observed in evolved diploids (S1B Fig). These results suggest that the activities regulated by TORC1 were altered in these evolved tetraploid clones.

To ask if the TORC1 network has been changed in the evolved tetraploid cells, we first examined the *TOR1* gene, which encodes a major component of TORC1. However, we did not find obvious changes in the expression level or gene sequence. Previously, our transcriptome data had already indicated that many genes involved in the stress response, ribosome biogenesis and translation changed their expression levels in evolved cells. This prompted us to examine the downstream regulators, especially the Sch9 protein, whose activity is regulated by TORC1 and other signaling pathways and is involved in regulation of ribosome biogenesis and translation initiation [42]. An HA tag was fused to one copy of the *SCH9* gene in the ancestral diploid, tetraploid and all evolved tetraploid clones, and Western blotting was used to detect the levels of Sch9-HA protein. Sch9 had increased by more than 1.5-fold in most evolved clones compared with ancestral tetraploids (Fig 5C). Interestingly, before experimental evolution, the relative abundance of Sch9 in tetraploids was about 1.8-fold lower than that in diploids ((2N-Sch9-HA/2N-G6PDH)/2×(4N-Sch9-HA/4N-G6PDH) = 1.76 ± 0.09; Fig 5D). These results strongly suggest that evolved tetraploids had adjusted their physiology to become more diploid-like. However, we did not observe obvious changes in the mRNA level of *SCH9*, suggesting that the evolved cells had increased the protein stability or translation efficiency of Sch9. To further delineate the mechanism, we performed a cycloheximide chase assay to examine the protein stability of Sch9. Cells from a representative evolved clone (evo4N-4) and its ancestor were treated with cycloheximide to stop protein synthesis and the degradation rate of Sch9-HA was measured. As shown in Fig 5E, the half-life of the Sch9 protein in the ancestral and evolved clones was 6.56 min and 12.01 min, respectively. This result suggests that the increased Sch9 abundance observed in evolved clones may result from enhanced protein stability of Sch9.

The TORC1 pathway and Sch9 have been shown to influence cell size [51, 53]. We examined the cell size of evolved tetraploid and diploid clones. Although all evolved tetraploids exhibited reduced cell size, a similar trend was observed in evolved diploids (S3 Fig), suggesting that this is probably a general outcome for evolving populations.

### Overexpressing *SCH9* enables ancestral tetraploid cells to exhibit evolved phenotypes

**Fig 6:**
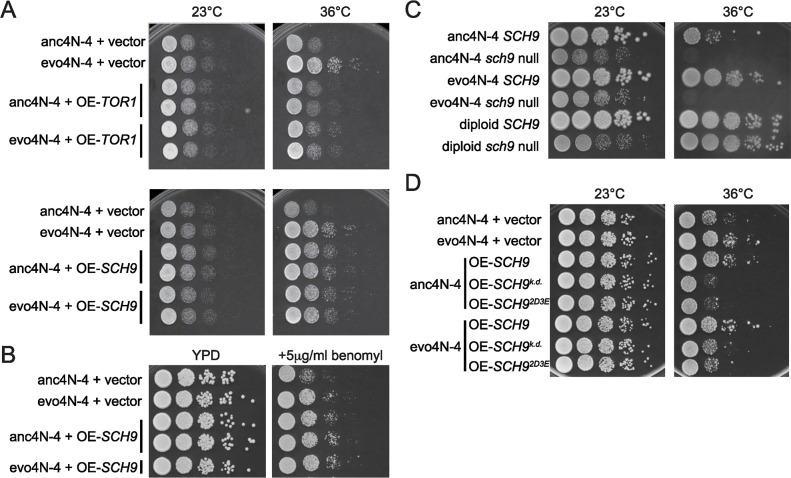
Sch9 plays a key role in the evolved phenotype. (A) Overexpressing *SCH9*, but not *TOR1*, enhanced the growth of anc4N-4 cells to a level near evo4N-4 cells. Multicopy plasmids carrying either *TOR1* or *SCH9* (OE-*TOR1* and OE-*SCH9*) were introduced into the anc4N-4 and evo4N-4 strains and tested for their effects on cell growth at 36°C. Overexpression of *TOR1* had no effect on ancestral cells and negative effects on evolved cells. Overexpression of *SCH9* had no effect on evolved cells but enhanced the growth of ancestral cells. (B) Ancestral tetraploids with *SCH9* overexpression exhibited improved fitness on plates containing the microtubule depolymerizing drug benomyl. (C) The enhanced survival of evolved tetraploids at 36°C was completed abolished in the absence of Sch9. Two premature stop codons were introduced into all genomic copies of *SCH9* to generate the *sch9* null mutants (see Materials and Methods). For each strain, we performed phenotypic assays for at least three *sch9* null mutant clones and observed consistent phenotypes between clones. (D) Both activity and regulation of the kinase are important for the evolved phenotype. Overexpression of kinase-dead (*SCH9*^*k*.*d*.^) or constitutively active (*SCH9*^*2D3E*^) mutants showed no effect at 23°C, but reduced the growth of evolved cells at 36°C. The correct ploidy was confirmed for all strains used in this figure using flow cytometry.

Although our data provides a link between down-regulated ribosome biogenesis and translation, and altered Sch9 regulation, it is still unclear how much Sch9 or the TORC1 network contributes to the evolved phenotype. To address this question, we introduced multicopy plasmids carrying either *SCH9* or *TOR1* (*pRS426-SCH9* and *pRS426-TOR1*) into the anc4N-4 and evo4N-4 strains and tested their effects on cell growth at 36°C. Overexpressing *SCH9* enhanced the growth of anc4N-4 cells to a level near that of evo4N-4 cells (Fig 6A). However, the presence or absence of *pRS426-SCH9* did not cause differences in the growth of evo4N-4 cells, suggesting that the growth-enhancing effect of *SCH9* might have reached a maximum in the evolved cells. In addition, overexpression of *SCH9* did not have any effect on diploid cells (S4A Fig), indicating that it is a tetraploid-specific effect. Interestingly, overexpression of *TOR1* did not improve the growth of anc4N-4 cells at 36°C. Moreover, it mildly reduced the growth of evo4N-4 cells. These results suggest two possible explanations for the evolved phenotype: it is due to the changes in the activities of other molecules that regulate Sch9, or it results from changes in the Sch9 branch of the TORC1 network, rather than the entire pathway. Previously we observed that evolved clones were more resistant to the microtubule depolymerizing drug benomyl. We tested whether Sch9 was also involved in this evolved phenotype. Ancestral tetraploids carrying *pRS426-SCH9* exhibited improved fitness on benomyl-containing plates, suggesting that Sch9 contributed to both improved growth at high temperatures and resistance to benomyl (Fig 6B).

### Sch9 plays a key role in the evolved phenotype

Tetraploid cells are notorious for their difficulty in genetic manipulation due to the copy number of genes and instability of the genome. To directly examine the effect of Sch9, we employed the CRISPR/Cas9 system to generate the *sch9* null mutants in tetraploid cells (see Materials and Methods) [54–57]. The *sch9* mutant cells already exhibited slow growth phenotypes at 23°C. When challenged with high temperatures (36°C), the enhanced survival of evolved tetraploids was completely abolished in the absence of Sch9, suggesting that Sch9 is crucial for the evolved phenotypes (Fig 6C).

The Sch9 activity is subjected to complicated regulation [58]. To further understand how Sch9 contributed to the evolved phenotypes, we examined the fitness of the evolved clone with different *SCH9* mutants. A dominant negative kinase-dead mutant (*SCH9*^*k*.*d*.^) was introduced into evo4N-4 and anc4N-4 cells [59], and then the cell growth was measured at both 23°C and 36°C. Overexpression of the *SCH9*^*k*.*d*.^ mutant did not have any obvious effect at 23°C, but it interfered with the cell growth of evolved tetraploids at 36°C (Fig 6D). In contrast, overexpressing *SCH9*^*k*.*d*.^ in diploids had no effect on cell growth at high temperatures (S4B Fig). These results demonstrated that the kinase activity of Sch9 is required for tetraploid cells to grow at higher temperatures. We tested a constitutively active mutant (*SCH9*^*2D3E*^) to see whether it could generate even stronger evolved phenotypes [42]. Intriguingly, cells carrying this mutant exhibited defects similar to the kinase-dead mutant (Fig 6C). In the *SCH9*^*2D3E*^ mutant, five TORC1-dependent phosphorylation sites have been mutated to acidic residues (aspartic acid or glutamic acid) to mimic the phosphorylated active state which allows cells to become rapamycin resistant. However, such mutations could also abolish regulation of Sch9 activity [42]. One possible explanation for our observation is that both the activity and regulation of Sch9 are important for the evolved phenotypes. Further experiments will be needed to clarify this issue. Nonetheless, both kinase-dead and constitutively active mutants exerted no effect on diploid cells (S4B Fig), indicating that tetraploid and diploid cells have different requirements or regulation of Sch9.

In our transcriptome data, the major group of genes with increased mRNA levels in evolved tetraploids was involved in protein folding. In addition, all the evolved clones showed significant improvements when grown at high temperatures. We investigated the possible role of molecular chaperones in the evolved phenotypes by overexpressing two different types of heat shock proteins, Hsp42 (a small heat shock protein) and Ssa4 (a member of the Hsp70 family). However, overexpression of *HSP42* or *SSA4* in ancestral tetraploid cells did not lead to evolved phenotypes, suggesting that evolved phenotypes may not simply result from enhanced stress resistance (S5 Fig).

### Evolved tetraploids can maintain their genomes in non-optimal conditions

**Fig 7:**
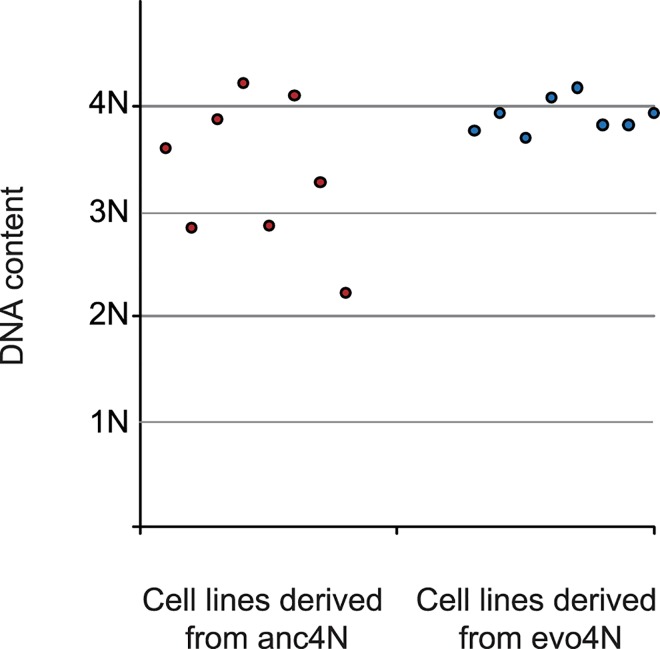
Evolved tetraploids can stably maintain their genomes when growing under non-optimal conditions. Eight ancestral tetraploid clones (anc4N-1 to anc4N-8) and eight evolved tetraploid clones (evo4N-1 to evo4N-8) were propagated in regular YPD medium at 30°C for 200 generations and the evolved cultures were analyzed using flow cytometry. Half of the cell lines derived from ancestral clones became aneuploid or diploid, but all cell lines derived from evolved clones remained tetraploid.

It has previously been observed that newly-constructed tetraploids quickly become aneuploids or diploids within a few hundred generations when propagated at 30°C [40]. If our evolved clones had already fixed the major problems caused by genome duplication, they should not only have higher fitness, but also have higher genome stability under non-optimal conditions. Eight evolved tetraploid clones and eight ancestral tetraploid clones were propagated in regular YPD medium at 30°C with daily dilution. The same numbers of evolved and ancestral diploid clones were set up as the control. After evolving for around 200 generations, the evolved populations were analyzed using flow cytometry. In the populations derived from ancestral tetraploid clones, three of them became aneuploid and one turned diploid. In contrast, all the populations derived from evolved tetraploid clones remained tetraploid and all control populations remained diploid (Figs 7 and S6). These results provide direct evidence that the first round of evolution in a less stressful condition (i.e. 23°C in our study) enabled tetraploid cells to stabilize their newly-formed genomes without drastic reorganization. Such stabilization might open new routes for further evolution of the tetraploid cells.

## Discussion

Polyploidization events have been repeatedly observed in different kingdoms of life with important consequences [21, 60–65]. In plants, multiple rounds of whole-genome duplication in angiosperms result in the expansion of the crucial developmental and regulatory gene repertoire [63, 66]. In yeasts and ciliates, whole-genome duplication events lead to the rapid emergence of many new species [21, 67]. Similar effects of polyploidization have also been implicated in vertebrates [20, 68, 69]. Although newly-formed polyploid genomes are intrinsically unstable and often degenerate into aneuploidy or diploidy [12, 17], recent genome analysis data reveal that, in many whole-genome duplication (WGD) events, large-scale genome rearrangement or chromosome loss do not immediately happen after genome duplication [19, 36, 62, 70, 71]. The best example can be found in the *Saccharomyces* complex in which most post-WGD species still maintain 16 chromosomes (derived from 8 ancient chromosomes), despite that many chromosomal rearrangements have occurred in the past 100 million years [72, 73]. How do cells stabilize a newly-duplicated genome at the early stage of polyploidy evolution? Our current data provide some clues for this long-standing enigma. By adjusting protein homeostasis through Sch9, cells are able to adapt to the polyploid genome and maintain it even under stressful conditions. More complicated adaptation may follow using the extra copies of genetic material.

Sch9 is a highly conserved protein kinase of the AGC kinase family [42, 74, 75]. By integrating different signaling modules, Sch9 regulates a broad range of growth-related cellular process [44, 76–81]. Many tetraploid-specific phenotypes, such as enlarged cell size, up-regulation of ribosome biogenesis, high sensitivity to heat, and a shortened chronological life span, are all reminiscent of the phenotypes observed in cells with altered Sch9 activities [59, 78, 82, 83]. Therefore, it is interesting to find that evolved cells adjust the protein level of Sch9 to make tetraploids become physiologically more diploid-like despite maintaining a tetraploid DNA content.

However, the more intriguing observation is that overexpression of Tor1 (the major component of TORC1) and Sch9 (one of the major regulators downstream of TORC1) show opposite effects. The protein abundance of Sch9 increased in most evolved clones, and overexpression of *SCH9* allowed ancestral tetraploids to exhibit evolved phenotypes. In contrast, overexpression of *TOR1* had no effect on ancestral tetraploids and showed negative effects on evolved cells. Earlier studies have shown that, in yeast and mammalian cells, complex negative and positive feedback loops exist between Sch9 (or the mammalian Sch9 homolog, S6K1) and the TORC1 network [43, 84, 85]. In addition, some other compensatory pathways may be turned on when Sch9 or TORC1 is compromised [78, 86]. In ancestral tetraploids, Sch9 is downregulated compared to normal diploids. It is possible that cells turn on other pathways to compensate the deficiency, leading to overexpression of many genes and a complicated transcriptome profile. In evolved tetraploids, Sch9 reverts to normal levels so cells display a diploid-like physiology and gene expression pattern (Figs 3 and 4). Alternatively, evolved phenotypes may result from changes in the upstream regulators of Sch9 other than TORC1. Earlier studies have indicated that Sch9 can be phosphorylated and regulated by other TORC1-independent signaling pathways [58, 87–90]. Further investigations will be required to elucidate the detailed underlying mechanisms.

Given the many challenges faced by a newly-formed polyploid genome, adjustments of Sch9 and its upstream regulators may only represent an initial step in the establishment of stable polyploids. For instance, studies in plants indicate that modifications of the meiosis machinery are also important for stabilizing polyploid genomes during sexual reproduction [91, 92]. Nevertheless, our ongoing laboratory evolution experiment has shown that the evolving lines can maintain tetraploid-like genomes even after 5000 generations at 23°C, suggesting that these cells have overcome the initial hindrance of genome instability (S7 Fig). Interestingly, when we examined the data of the same plant study that found evidence of selection in a stabilized polyploid species, four genes involved in the TORC1 network (TOR, AGC1.5, RAPTOR2 and AT3G19940) were found to show similar selective signatures [92]. Moreover, overexpression or mutations in components of TORC1 and the mammalian Sch9 homolog S6K1 are often observed in cancer cells, which are invariably aneuploid or polyploid [93–98]. These observations suggest that regulation of the *SCH9* orthologs or TORC1 may be critical for polyploid plant and animal cells.

In the past, large-scale chromosomal deletions or duplications in polyploid cells have been implicated in rewiring regulatory networks and inventing novel phenotypes [7, 99, 100]. While such global genome reorganizations allow cells to quickly respond to new environmental stresses, they also engender critical physiological challenges for cells [101]. Compared to euploids, aneuploids are often unstable and have reduced fitness in normal conditions, even though they confer beneficial effects under specific environments [97, 102, 103]. More importantly, aneuploidy may represent an evolutionary dead-end in a sexual population [104]. Meiosis of aneuploid cells generates gametes with different combinations of chromosomes that may result in reduced viability or fitness of gametes and zygotes [105]. Severe aneuploidy can cause complete sterile progeny [12, 106]. Stabilized polyploidy probably represents a critical prerequisite for long-term evolution of the population. Even if a stabilized polyploid genome does not provide immediate adaptive benefits to the cells, the extra copies of genetic material will grant cells more flexibility for their future adaptation.

Using laboratory evolution experiments, we have shown that changes in the activity of a conserved protein kinase, Sch9, stabilize newly-formed tetraploids. These stabilized tetraploids will now enable us to dissect their evolutionary trajectories under different selective regimes. Comparing the stabilized and newly-formed (and unstable) polyploids will provide further insights into the enigma of polyploidy evolution.

## Materials and Methods

### Strain constructions and experimental evolution

Two *S*. *cerevisiae* haploid strains (*MAT***a** and *MAT*α *ura3-1 ade2-1 his3-11*, *15 leu2-3*, *112 trp1-1 can1*::*MFAp-HIS3-MF*α*p-LEU2*) derived from the W303 background were used to generate isogenic diploid and tetraploid strains. In the haploid strains, the *SPO11* gene was first deleted using the kanMX6 or cloNAT deletion cassette. These diploid strains were transformed with the *pGAL1-HO-hphMX6* plasmid in order to switch the mating type from **a**/*α* to **a**/**a** or *α*/*α*. Tetraploid ancestral strains were generated by mating between **a**/**a** and *α*/*α* diploid strains.

Eight independent diploid and tetraploid colonies were chosen for the 1st round of evolution experiments. Diploid and tetraploid cells were propagated in 3 ml YPD (Yeast extract-Peptone-Dextrose) medium at 23°C through a daily 1000-fold dilution (about 10 generations). Evolving lines were alternatively marked with the kanMX6 or cloNAT drug resistant marker so any cross-contamination between cultures could be easily detected. Once every 10 transfers (around 100 generations) population samples from each line were stored in 20% glycerol at −80°C for later analysis. After evolving for 1000 generations, five single colonies were isolated from each line and their DNA contents were first confirmed (i.e. that colonies from tetraploid lines were still tetraploid) using flow cytometry. Next, the fitness of these colonies was measured using competitive relative fitness assays. The colonies with the highest fitness from each line were used for phenotypic characterizations. The laboratory evolution experiment had been continuing for 5000 generations at the time of manuscript preparation.

In the second round of evolution experiments, eight ancestral clones and eight evolved clones (isolated from the eight previously evolving lines at generation 1000) were propagated in 3 ml YPD medium at 30°C through a daily 1000-fold dilution. After evolving for 200 generations, the DNA content of all evolving lines was analyzed using flow cytometry.

### Flow cytometry analysis of DNA content

Flow cytometry analysis was used to determine the DNA content of ancestral and evolved cells. Total 5×10^6^ log phase cells were harvested, resuspended in ice-cold fixation buffer (40% ethanol, 0.1 M sorbital and 5 mM EDTA), and then kept at -20°C overnight. The fixed cells were then washed twice with 1 ml ddH_2_O and then washed with 1 ml PBS + 0.5% Triton X-100. After washing, the cells were treated with 0.5 ml 50 mM Tris-Cl (pH 8.0) with 150 μg/ml RNase A and incubated at 37°C for 16–18 hours. SYTOX® Green nucleic acid stain (Invitrogen Corp., Carlsbad, CA) and 38 mM sodium citrate were mixed in a 1:800 ratio and 300 μl of the mixture was added into the cell solution. The stained cells were diluted into 1 ml 0.1 M EDTA and sonicated for at least 3 minutes prior to flow cytometric analysis. A total of 10,000 cells were scored for DNA content using the BD FACScan system (Becton Dickinson, Franklin Lakes, NJ).

### Competitive relative fitness assay

The growth rates of individual colonies were measured using a flow cytometry-based competitive assay. A diploid strain carrying the Cwp2-YFP (yECitrine) fusion protein was used as the reference strain. Tested cells and reference cells were inoculated in YPD medium individually and acclimated for 24 h. The cells were subsequently diluted and refreshed in new media for another 4 h. Because the test tetraploid cells had a slower growth rate, reference and test cells were then mixed in a 1:3 ratio, diluted into fresh medium at a final cell concentration of 5×10^4^ cells/ml, and allowed to compete for 16 hours under indicated conditions. The ratio of the two types of cells was determined at the initial and final time points of the competition using the BD FACScan system (Becton Dickinson). These ratios were used to calculate the growth rate of the test strain. For each fitness measurement, at least three independent replicates were performed.

### Spot plate assay

Cells were grown in YPD medium at 23°C overnight. The cell cultures were serially diluted and spotted onto YPD plates with or without drugs (i.e. rapamycin or benomyl). The plates were then incubated at the indicated temperatures until colonies were easily observed.

### Array-based comparative genomic hybridization

Genomic DNA from ancestral and evolved clones was hybridized to microarrays to examine the chromosome copy numbers. Yeast oligoarrays (GPL7305) were produced at the Genomics Core, Institute of Molecular Biology, Academia Sinica using an Omnigrid 100 microarrayer (Digilab, Holliston, MA) and the Yeast Genome Array-Ready Oligo Set (Version 1.1, Operon, Huntsville, AL). The arrays were printed according to the protocol of the Institute of Molecular Biology Genomics Core (http://www.imb.sinica.edu.tw/mdarray/methods.html). Yeast genomic DNA was extracted using the Qiagen Genomic-Tip 100/G kit (Qiagen, Valencia, CA). Probe preparation and hybridization were performed as described [107]. The array data were analyzed using GeneSpring GX (Agilent, Santa Clara, CA).

### Whole-genome expression analysis

For the whole-genome gene expression analysis, test and reference clones were individually inoculated in rich medium and acclimatized for 24 hours. The cells were diluted and refreshed in new medium at 23°C or 36°C for another 4 to 6 hours, respectively. A total of 3×10^8^ cells were then harvested. Total RNA was isolated using Qiagen RNeasy Mini Kit (Qiagen). Probe preparation and hybridization were performed as described [107], and yeast oligoarrays (GPL7305) were used. The array data were analyzed using GeneSpring GX (Agilent). We excluded data with hybridization intensities lower than 500 as they were close to background values (< 100). The intensities of each array were log2-transformed and normalized using a Locally Weighted Scatterplot Smoothing (LOWESS) function [108]. Only if a change in gene expression was larger than 2-fold with a p-value smaller than 0.005 were the genes classified as differentially expressed.

Gene ontology (GO) analysis was performed using the integrated database FunSpec [109] with Bonferroni correction and a p-value threshold less than 10^−5^. Overlap statistics often employed to assess these associations do not correct for the overestimation of significance of gene sets with a very high number of annotations. To correct for this bias, we implemented Annotation Enrichment Analysis [110], which accounts for non-uniformity in annotations (with a p-value threshold less than 0.05). Enrichment scores were calculated by dividing the proportion of differentially-regulated genes classified into the indicated category by the proportion of those in the genome. GO categories are shown in Fig 4A and S2 Table only if they had at least 2-fold enrichment and included at least three genes.

### Growth rate measurement with rapamycin

Log-phase cells (OD_600_ = 0.4–0.6) were inoculated in the YPD medium with or without 10 ng/ml of rapamycin (which is an inhibitor of TORC1) and grown with an initial OD_600_ of 0.1 in 96-well tissue culture plates. The growth assays were performed at 23°C for 24 hours using Infinite 200 series plate readers (Tecan, Mannedorf, Switzerland) with continuous shaking. The OD values of the cultures were simultaneously recorded by built-in Magellan software. Doubling time and growth rate analyses were later performed using the OD values collected at different time-points spanning 24 hours.

### Protein extraction and quantification

Total protein was extracted by using an NaOH-lysis method for measuring Sch9 levels [111]. About 1×10^8^ cells were resuspended in 1 ml lysis buffer (0.185 M NaOH, 0.75% β-ME) and kept on ice for 10 minutes. Trichloroacetic acid (TCA) was added to the lysis buffer at a final percentage of 8% to concentrate proteins for electrophoresis. After 15 minutes, all residual supernatant was removed and the precipitant was resuspended in HU sample buffer (8 M Urea, 5% SDS, 0.2 M Tris-HCl pH 6.5, 1 m M EDTA, 0.01% bromophenol blue) supplemented with 2 M Tris base in a 50:3 ratio. The protein lysate was then incubated at 65°C for 10 minutes. Total protein lysate was separated by 6% SDS-PAGE, and blotted onto PVDF membrane (Immobilon-PSQ, Millipore, Billerica, MA) in a transfer buffer (25 mM Tris base, 200 mM glycine, 20% methanol). Membranes were blocked in 1% casein (Sigma C5890) blocking solution. Mouse monoclonal anti-hemagglutinin (anti-HA) antibody (Covance, Princeton, NJ) was used to detect HA-tagged proteins. G6PDH was used as an internal control protein and was detected by rabbit polyclonal anti-G6PDH antibody (Sigma A9521). For the HA-tagged construct, at least three independent transformants from each strain were tested to ensure that consistent patterns were observed between clones.

### Quantitative PCR

Total RNA was isolated from cells using the Qiagen RNeasy Mini Kit (Qiagen). First-strand cDNA was synthesized for 2 h at 37°C using the High Capacity cDNA Reverse Transcriptase Kit (Applied Biosystems, Foster City, CA). A 20-fold dilution of the reaction products was then subjected to real-time quantitative PCR using gene-specific primers, the Fast SYBR^®^ Green master mix, and an Applied Biosystems 7500 Fast Real-Time PCR System (Applied Biosystems). Data were analyzed using the built-in analysis program.

### Cycloheximide chase assay

The ancestral and evolved tetraploid *SCH9-HA* strains were grown in the YPD medium for 16 hours and then refreshed to log phase (OD_600_ = 0.4–0.6) at 23°C. Then, 35 μg/ml of cycloheximide was added to the culture (defined as the 0 min time-point). Cells were collected at different time points (t = 0, 10, 20, 30, 40 and 50 min) and frozen at −80°C. After all samples were collected, total protein was extracted and Sch9-HA levels were examined by immunoblotting. The relative intensity of Sch9-HA was calculated as the ratio of Sch9-HA to G6PDH protein abundance at each time-point using the ImageJ software. All the data points were fitted to the function ln(P_t_/P_0_) = [ln(1/2)/T_1/2_]t, in which P represents the relative intensity of Sch9 and T_1/2_ represents the half-life. The degradation constant [k = ln(1/2)/T_1/2_] was estimated as the slope of this linear regression line on the ln(P_t_/P_0_)-t plot.

### CRISPR-Cas directed *SCH9* mutagenesis in tetraploid cells

The CRISPR-Cas system was modified from the previous methods [54, 55]. The gRNA expression plasmid (pRS426-gRNA.CAN1.Y) was obtained from Dr. George Church [54] and the target sequence of *SCH9* (AAATCGTCGAATCAGGATACTGG, with the PAM sequence underlined) was designed using the online tool CRISPy (http://staff.biosustain.dtu.dk/laeb/crispy_yeast/) [56]. The gRNA expression plasmid was first linearized by cutting at the NheI and KpnI sites and the following two pairs of PCR primers were used to generate the gRNA insert fragment,

gRNA-F1: ACCGTATTACCGCCTTTGAG,

gRNA-R1: AAAACGTATCCTGATTCGACGATTTGATCATTTATCTTTCACTGCGGAG,

gRNA-F2: CGTCGAATCAGGATACGTTTTAGAGCTAGAAATAGCAAGTTAAAATAAG,

gRNA-R2: GAAGGGAAGAAAGCGAAAGG.

The donor DNA was used to introduce two premature stop codons (bp 40 to 45) into the *SCH9* coding region and it also destroyed the PAM sequence to prevent repeatedly targeting by Cas9. The donor DNA was generated using the following two pairs of PCR primers,

donor DNA-F1: ACCCACTCTCACATAATCACC,

donor DNA-R1: GTTGGTGTTGAGAGCTAAACTATCAATCCTGATTCGACGATTTTGATG,

donor DNA-F2: CGTCGAATCAGGATTGATAGTTTAGCTCTCAACACCAACATCC,

donor DNA-R2: CCTGACTAATAGCGGTGGTCG.

The original plasmid carrying the high specificity Cas9 nuclease, eSpCas9(1.1), was obtained from Addgene (plasmid #71814) and the enzyme was further subcloned into the yeast pRS414 vector in our lab [57].

For constructing the *sch9* null mutant, diploid and tetraploid cells were first transformed with the pRS414-eSpCas9(1.1) plasmid. The transformants were then transformed with the DNA mixture of the linearized gRNA plasmid (0.12 μg), gRNA insert fragment (0.3 μg) and donor DNA (0.6 μg). For each strain, single colonies were picked and checked by PCR using wild-type-specific and mutant-specific primers. Only the colonies carrying mutant *sch9* were further examined using the Sanger sequencing. The correct clones were propagated in nonselective YPD medium to lose the plasmids and their ploidy was confirmed using flow cytometry. For each strain, we performed phenotypic assays for at least three *sch9* null mutant clones and observed consistent phenotypes between clones.

### Data access

The CGH array data are available at the NCBI Gene Expression Omnibus (GEO) (http://www.ncbi.nlm.nih.gov/geo/) under the accession number GSE76595. The expression data are available at GEO under the accession number GSE 76596.

## Supporting Information

S1 Fig Evolved diploid clones do not alter their fitness significantly at high temperature or resistance to rapamycin. (A) Ancestral and evolved diploid clones were serially diluted and spotted onto YPD plates. The plates were then incubated at 23°C, 36°C or 38°C until colonies were easily observed. No single colonies were found on the plate incubated at 38°C, even after one week. (B) Ancestral and evolved diploid cells were serially-diluted and spotted onto YPD plates containing different concentrations of rapamycin (0, 5, 10 and 20 ng/ml). The plates were then incubated at 23°C until colonies were easily observed.
(EPS)

S2 Fig Tetraploid cells become diploid-like in their gene expression after evolution. Expression levels of all yeast genes of anc4N-4 and evo4N-4 were compared to that of ancestral diploids, and a higher correlation in the expression level was observed between ancestral diploids and evolved tetraploids.
(EPS)

S3 Fig Cell size is reduced in all evolved clones. Cells from evolved tetraploid clones (A) and diploid clones (B) were measured for their cell size using flow cytometry. All the measurements were normalized to the cell size of ancestral clones.
(EPS)

S4 Fig Overexpression of *SCH9* does not have an obvious effect on the growth of diploid cells at high temperatures. (A) Unlike tetraploid cells, diploid cells with or without overexpression of *SCH9* and *TOR1* exhibited similar growth rates at 38°C. (B) Overexpression of kinase-dead (*SCH9*^*k*.*d*.^) or constitutively active (*SCH9*^*2D3E*^) mutants does not affect the growth of diploid cells at high temperatures.
(EPS)

S5 Fig Overexpression of *SSA4* or *HSP42* does not improve the growth of ancestral tetraploid cells at high temperatures. Multicopy plasmids carrying either *SSA4* or *HSP42* (OE-*SSA4* and OE-*HSP42*) were introduced into the anc4N-4 and evo4N-4 strains and tested for their effects on cell growth at 36°C.
(EPS)

S6 Fig Evolved diploids do not change their ploidy after evolving at 30°C for 200 generations. Eight ancestral diploid clones (anc2N-1 to anc2N-8) and eight evolved diploid clones (evo2N-1 to evo2N-8) were propagated in regular YPD medium at 30°C for 200 generations and the evolved cultures were analyzed using flow cytometry. All the lines remained diploid.
(EPS)

S7 Fig Flow cytometry analysis indicates that the DNA content of evolved cultures is stably maintained even after 5000 generations of laboratory evolution. Eight newly-constructed isogenic tetraploid cells were propagated in YPD medium at 23°C with daily dilutions. Cell cultures at generation zero (ancestral), 1000, 2000, 3000, 4000 and 5000 were analyzed using flow cytometry.
(EPS)

S1 Table Differentially-expressed genes between evolved and ancestral cells at 23°C and 36°C. Only genes with expression changes greater than two-fold (p < 0.005) are included. In the diploid data set, only one gene passed this criterion.
(XLSX)

S2 Table GO analysis of misregulated genes of the evo4N-4 shown in Fig 4A, along with p-values corrected for biases due to non-uniformity in annotations. (XLSX)

S3 Table Comparison of differentially-expressed genes from rapamycin-treated cells and evo4N-4 cells grown at 36°C. Data for the rapamycin-treated cells were taken from the study by Hardwick et al. [52]. Overlapping genes are labeled in red.
(XLSX)
